# Patients and relatives as auditors of safe practices in oncology and hematology day hospitals

**DOI:** 10.1186/s12913-020-06018-3

**Published:** 2021-01-07

**Authors:** Isabel Rodrigo Rincón, Isabel Irigoyen Aristorena, Belén Tirapu León, Nicolás Zaballos Barcala, Maite Sarobe Carricas, Joaquín Lobo Palanco, María Luisa Antelo Caamaño, Marta Patricia Martin Vizcaíno, Susan Burnett

**Affiliations:** 1 Complejo Hospitalario de Navarra, Servicio Navarro de Salud – Osasunbidea, REDISSEC, IdiSNA, Pabellón G. Irunlarrea, 3, 31008 Pamplona, Spain; 2 Servicio de Apoyo a la Gestión Clínica y Continuidad Asistencial, Complejo Hospitalario de Navarra / IdiSNA, Pamplona, Spain; 3 Servicio de Anestesia y Reanimación, Complejo Hospitalario de Navarra, Pamplona, Spain; 4 Servicio de Farmacia, Complejo Hospitalario de Navarra, Pamplona, Spain; 5 Servicio de Cuidados Intensivos, Complejo Hospitalario de Navarra, Pamplona, Spain; 6 Department of Surgery & Cancer, Medical School, Faculty of Medicine, Imperial College London, St Mary’s Campus, London, UK

**Keywords:** Patient safety, Audit and feedback, Safe practices, Patient-centred care

## Abstract

**Background:**

When there is a gap in professionals’ adherence to safe practices during cancer treatment, the consequences can be serious. Identifying these gaps in order to enable improvements in patient safety can be a challenge. This study aimed to assess if cancer patients and their relatives can be given the skills to audit reliably four safe practices, and to explore whether they are willing to play this new role.

**Methods:**

We recruited 136 participants in 2018, from the oncology and haematology day hospital of a tertiary hospital in Spain. Patient identification, hand hygiene, blood or chemotherapy identification, and side effects related to transfusion and chemotherapy, were the safe practices selected for evaluation.

The study comprised two parts: an interventional educational program and a cross-sectional design to collect data and assess to what degree participants are able and willing to be auditors depending on their characteristics using multivariate logistic regression models. A participant’s auditing skill were assessed pre and post the educational intervention.

**Results:**

The model was seeking predictors of being a good auditor. 63 participants (46.3%) were classified as good auditors after the training. To have younger age, higher educational level and to have had an experience of an adverse event were associated with a higher probability of being a good auditor. Additionally, 106 (77.9%) participants said that they would like to audit anonymously the professionals’ compliance of at least three of four safe practices. The willingness to audit safe practices differed depending on the safe practice but these differences did not reach statistical significance.

**Conclusions:**

The data gathered by patients and relatives acting as auditors can provide healthcare organizations with valuable information about safety and quality of care that is not accessible otherwise. This new role provides an innovative way to engage patients and their families’ in healthcare safety where other methods have not had success. The paper sets out the methods that healthcare organizations need to undertake to enrol and train patients and relatives in an auditor role.

## Background

Improving patient safety is one of the major targets for healthcare organizations. Studies have shown that adverse events in hospitals can vary between 3 to 17% of all hospital admissions [1–3]. World Health Organization (WHO) safety programme and Spanish Ministry of Health safety programme; as well as other European organizations like the European Patient Safety Foundation (EPSF), medical societies and healthcare providers, encourage the development tools to ensure that healthcare is both safe and patient-centered [4]. There has been quite a lot of work to engage patients in safety, for example in medication safety. The WHO Alliance for Patient Safety also emphasized that the patients’ family members could play an important role in the improvement of care. Nevertheless, despite the emphasis and importance of patients’ involvement in promoting safety and reducing adverse events, there has been insufficient progress in this area [5, 6]. Retrospective chart audit studies of acute care in several countries have shown that patients experience one or more harmful adverse events while hospitalized and that about half of these events are preventable [7].

Cancer care is complex and requires particular effort to assure safety in care delivery for patients. It is known that cancer patients are vulnerable to breaches of safe practices because of their health conditions (for example immune suppression) and the nature and risks associated with their usual treatments. The areas of cancer care where gaps in adherence to safe practices can lead to adverse events with potentially serious consequences include patient identification; correct choice, dose and route for the delivery of chemotherapy and transfusion medication; and infection control.

Walsh et al. estimated the rate of error in the administration of chemotherapy as 8.2 per 1000 orders in oncology adult patients in the outpatient setting, causing damage in one error for every 1000 orders [8]. A considerable fraction of these occur in the phase of administration, which is observable by patients.

The National Haemovigilance Report published in Spain, in 2016, recorded 332 errors in the administration of blood products, and 32% of them happened at “the bedside of the patient.” [9]. However, studies on patients’ involvement in transfusion safety are scarce [10].

Between 5 and 10%, patients admitted to a hospital will develop at least one nosocomial infection. Hand hygiene is one of the main measures to prevent these infections [2]. However, according to international studies, the adherence of professionals to hand hygiene is less than 50% [11]. Multimodal strategies are being implemented to improve their adherence. These strategies have had a variable effectiveness (51–83%) and there is some evidence that patients can play an important role in improving the compliance [12].

Proper patient identification at every step of clinical care is vital to ensure patient safety. However, despite the priority placed on addressing this issue, significant problems persist. “Wrong patient,” “wrong site,” and “wrong procedures” continue to be among the most frequently submitted sentinel events reported to The Joint Commission in the USA [13].

Such data has the potential to allow clinical teams and services to consider the reasons for non-adherence and to make changes to improve patient safety.

Furthermore, it is well established that patients and their families (including friends and informal caregivers) have unique knowledge and are able to detect if their care is safe and patient-centred [6, 14, 15]. Patients and their families are present during the whole care episode and often are the only members who are aware of lapses in safety thus being a useful source of information about patient safety. Information gathered by patients and their families (P&Fs) gives healthcare organizations an opportunity to learn and improve the system of care [15–17]. One way of playing the auditor’s role is through the patient-as-observer approach [18, 19]. This approach involves recruiting a cohort of patients with multiple healthcare contacts who report on a continuous basis whether health professionals correctly follow patient safety protocols. However, there is little evidence collected directly from patients about their willingness or ability to be involved in this new patient safety role [11, 20].

The aims of this study were to assess if in controlled conditions, a) P&Fs can be given the skills and reliably audit safe practices; b) to determine the characteristics of good auditors; and c) to explore if P&F’s are willing to play this new role anonymously.

Based on the evidence from real-time safety audits performed during routine work, it is known that such audits can detect a broad range of errors [17]. From this it was considered that the patients’ participation in the role of an auditor could assist in identifying gaps in safety and this could lead to work to improve patient safety [6, 17, 21]. The safe practices selected for evaluation were

- patient identification,
- hand hygiene,
- blood or chemotherapy identification, and
- secondary effects of chemotherapy/transfusion.

These practices were selected as they can be observed by P&Fs and because of the serious risks to patients if the protocols are not followed.

## Methods

The methods involved an interventional educational program to improve the participants’ skills to audit safe practices and a cross-sectional study using a questionnaire to collect data on the participants’ perceptions about their willingness to be auditors. Navarra’s Department of Health Research Ethics Committee’s approval was obtained for the study (approval number: Pyto2015/62), and all participants provided written informed consent. Two focus groups were organized during the development phase of the study involving 33 patients who were not involved in the main study. The opinions of the patients in these focus groups were used to design the study including the development of the training methods and materials (sent for publication pending acceptance).

### Main study participants

A consecutive sample of 136 participants was recruited between March and October 2018 from the oncology and haematology day hospital of a tertiary hospital in Spain. Patients were eligible to participate if: they were older than 18 years; it was not their first treatment appointment; the treatment lasted several hours; the healthcare professionals in charge considered their physical and psychological status as acceptable for participation; and they were able and willing to give their informed consent to participate.

Patients’ family members were recruited after being informed about the study and providing their consent to participate. For this study, we considered as relatives not only family but also friends or informal caregivers. The research was conducted while patients were being treated or just immediately after being treated in order to emulate real conditions.

We selected day hospital oncology P&Fs because they have multiple contacts with the healthcare organization and thus it can be feasible to train them. Likewise, non-compliance of professionals with safe practices can have serious consequences for the patients’ health.

### Educational material production

The research materials comprised: training brochures, videos (assessment and evaluation), evaluation grids, and a questionnaire to assess P&Fs’ willingness to audit. The training brochures explained how healthcare professionals must implement the four safe practices selected for evaluation. The videos were filmed in the real places where patients receive treatment. There were two different stories, depending on the type of P&F. They showed a patient who goes to the day hospital to receive chemotherapy or to receive transfusion. Both videos were in a story-like format intending to show, in the most realistic way, the interaction between a patient and healthcare professionals during treatment. The right way to implement the safe practices was highlighted in the training video. The actors were different in all videos in order to make a distinction between the training and the assessment video. Additionally, the content of the materials used were adapted according to the treatment (oncology or haematology).

**Fig. 1:**
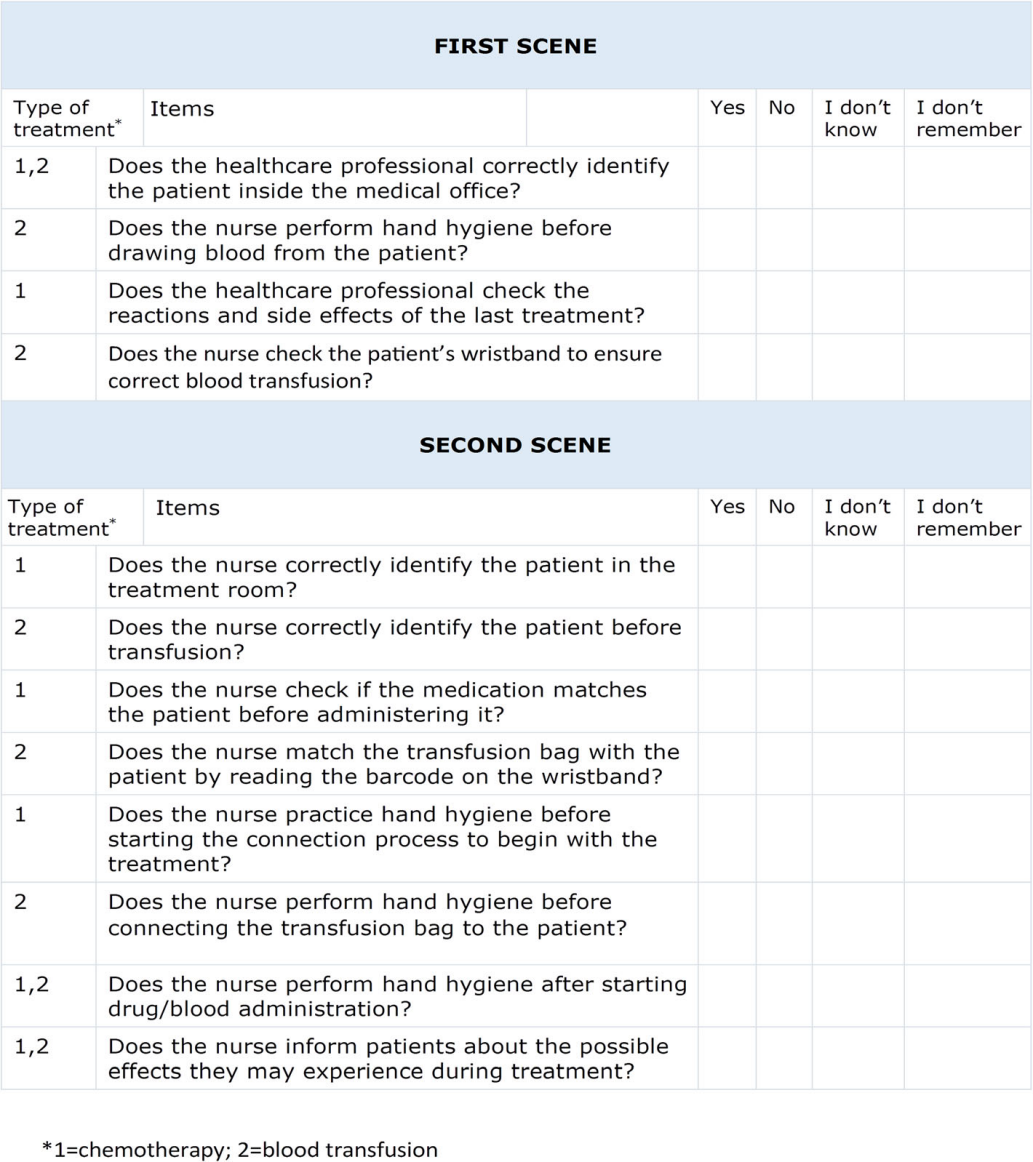
Items included in the evaluation grid

The evaluation grid had 7 or 8 questions depending on the treatment (Fig. 1). The questions dealt with the observation of the fulfilment of the four safe practices studied in the video.

All the materials were validated through testing with 48 convenience individuals (not involved in the main study) to ensure that a) all the materials were easily comprehensible and b) that the training enabled the individuals to detect the patient safety incidents in the videos.

The videos and training leaflets were produced in the hospital by personnel working on the study and volunteers that collaborate as actors in our medical installations. The digitization unit helped with video recording and editing.

### Procedure and data collection

Initially, the P&Fs watched an assessment video only once. Immediately after viewing it, they completed an evaluation grid. Then, they were provided with a training brochure. Afterwards, participants watched a training video. Participants could watch this last video as many times as they wished. After reading the material and watching the videos, the participants once again watched the assessment video and filled a second evaluation grid. Later, they completed the questionnaire about their willingness to become auditors.

The participants could ask questions during this process. All the videos were played on a tablet. The whole process lasted between 60 and 90 min. Depending on the time availability of the participants, debriefing (feedback) was done once the process had finished. Due to their time not all participants were able to take part in this feedback process. The benefit, or otherwise, of this debriefing was not studied here but is something that would be of interest in future research.

### Measures

A variable named “potential auditor” was created in order to analyse the P&Fs’ degree of willingness to audit healthcare professionals practice. Participants who answered that they would audit at least 3 out of 4 safe practices were considered potential auditors. A variable called “good auditor” was also created to measure participants who correctly answered more than 75% of the items. This cut-off point was chosen based on the margin of error that the organization was willing to assume.

The dependent variables were: “*potential auditor*” and “*good auditor.*” The independent variables were gender, age, type of participants, type of treatment, and number of healthcare encounters, adverse events suffered, education level, and general perception of hospital safety.

### Data analyses

Data were analysed using SPSS version 20.0 for Windows. Wilcoxon and McNemar tests were used to compare before-after results; Pearson’s chi-square test or Fisher’s exact test was used for discrete variables and Student’s t-test for continuous ones; and logistic regression (backstep) was used to measure the influence of different variables on P&Fs’ willingness to audit and being a good auditor. A *p* value < 0.05 was considered to be significant.

## Results

### Participant characteristics

In total, 136 P&Fs agreed to participate (63.0% response rate). The characteristics of the participants are set out in Table 1. These included age, gender, education level, number of health encounters during last year, type of participants, type of treatment, healthcare professionals, if they have suffered any adverse event, and perception about hospital’s safety.

**Table 1:** Participants’ characteristics

Socio-demographic variables		Total sample
		***N*** = 136 (%)
Age		Range 20–87 years
		(Mean 57.3, SD 13.8)
Gender		
	Male	64 (47.1)
	Female	72 (52.9)
Education^a^		
	Basic level	65 (47.8)
	Medium level	32 (23.5)
	High level	36 (26.5)
	Missing	3 (2.2)
Type of participant		
	Patients	90 (66%)
	Relatives	46 (34%)
Type of treatment		
	Chemotherapy	101 (74.3)
	Transfusion	35 (25.7)
Healthcare professional		
	Yes	14 (10.29)
	No	103 (81.74)
	Missing	19 (13.28)
Number of day hospital visits during the last 12 months		Median 6
		(Interquartile range: 3–14.5)
Number of hospital stays during the 12 months		Median 1
		(Interquartile range 0–2)
Adverse events	Yes	30 (22.1)
	No	106 (77.9)
General perception of hospital safety	Not safe enough	3 (2.1)
	Quite safe	72 (53.3)
	Absolutely safe	60 (44.4)

^a^Education level: basic level means no formal education or primary education, medium level means high school or vocational education and training diploma, and high-level means university degree and above

The proportion of patients participating (66.0%) was much higher than that of relatives because often patients come to the day hospital on their own. Almost half of the P&Fs had a basic level education (just primary and secondary school studies) and the others university or superior studies. The proportion of women and men was very similar. As expected, most P&Fs were not healthcare professionals although 14 were. The number of healthcare encounters (mean: 10 hospital visits and 1 hospital stay) indicated that the P&Fs had plenty of experience of visiting the hospital.

### Ability to recognize safe practices after training

Of the P&Fs, 88.6% answered that they felt confident in identifying 3 out of 4 practices after the training.

### Participants’ skills to be auditors

The variable “*good auditor”* was measured before and after the training to analyse the baseline skills of the participants to be auditors and the improvement achieved after the training. The overall percentage of this variable increased from 30.4% before training to 46.3% after it (McNemar *p* = 0.000). P&Fs had similar baseline skills (30.0% patients vs. 30.4% relatives). After the training, relatives improved more than did the patients (58.7% vs. 40.0%; p bilateral exact Fisher = 0.046).

### Characteristics of good auditors

Sixty-three participants (46.3%) were classified as “*good auditors”* after the training (% of correct answers > 75%). Age, education level, type of participants, type of treatment, and adverse events showed statistically significant differences in the bivariate analysis tests (Table 2).

**Table 2:** Characteristics of “good” and “poor” auditors from the bivariate analysis. Variables that predict being a “good” auditor from logistic regression analysis

Bivariate analysis of the P&Fs characteristics between “good” and “poor” auditors * Good auditors (> 75% correct answers) ** Poor auditors (= < 75% correct answers)					Logistic regression analysis of the variables that predict being a “good” auditor				
		Good auditors* *n* = 63	Poor auditors** *n* = 73	**p value**	**Beta**	**p value**	**Odds Ratio**	**95% CI**	
**Age: mean (sd)**		51.4 (12.5)	62.5 (12.7)	0.000	−0.061	0.001	0.940	0.908	0.974
**Gender: n (%)**	Female	33 (46.5)	39 (53.5)	0.903					
	Male	30 (47.6)	34 (52.4)						
**Education: n (%)**	Basic level	20 (31.7)	45 (68.2)	0.000		0.006	1 (referent)		
	Medium level	14 (43.8)	18 (56.3)		0.652	0.208	1.920	0.696	5.301
	High level	28 (77.8)	8 (22.2)		1.738	0.001	5.684	1.947	16.591
**Adverse events suffered n (%)**	No	22 (73.3)	8 (26.7)	0.001	0		1 (referent)		
	Yes	41 (38.7)	65 (61.3)		1.658	0.002	5.250	1.861	14.806
**General perception of hospital safety**	Totally safe	19 (31.7)	41 (68.3)	0.002					
	Other categories	43 (57.3)	32 (42.7)						
					2.388	0.027	10.894	**Constant**	
					R^2^ Nagelkerke = 0.411 X^2^ Hosmer and Lemeshow =3.530 Sig = 0.897				
**Type of participants: n (%)**	Companions	27 (60.0)	19 (40.0)	0.046					
	Patients	36 (40.4)	54 (59.6)						
**Type of treatment: n (%)**	Chemotherapy	52 (52.5)	49 (47.5)	0.05					
	Transfusion	11 (31.4)	24 (68.6)						
**Healthcare professional n** (%)	Yes	8 (57.1)	6 (42.9)	0.783					
	No	54 (53.5)	49 (46.5)						
**Number of Day Hospital visits: median (IQR)**		6 (15–3)	7 (12–3)	0.649					
**Number of hospital stays: median (IQR)**		1 (2–0)	0 (1–0)	0.108					

A multivariable logistic regression model was developed to simultaneously consider all the variables in order to predict the outcome of *“good auditor.”* The variables age, adverse events, and education level had statistically significant coefficients (see Table 2).

**Fig. 2:**
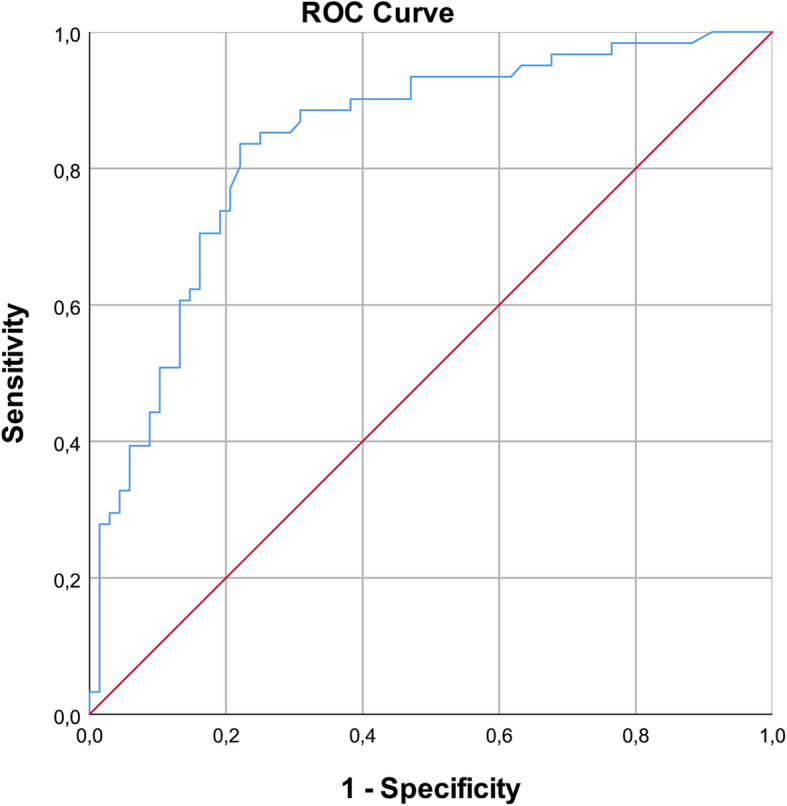
Receiver operating characteristic (ROC) curve of the variables that predict a P&Fs being a “good auditor”

Younger age, high educational level and experience of an adverse event are the characteristics that best predict a P&Fs being a *“good auditor”.* The value that indicates the area under curve was 0.838 indicating that the variables included in the model have a strong effect (see Fig. 2).

### Willingness to be an auditor

The percentage of willing and therefore “*potential auditors*” varied for each safe practice. It was 72.1% for hand hygiene, 75.0% for secondary effects of chemotherapy/transfusion, 79.1% for patient identification, and 80.1% for blood or chemotherapy identification. Moreover, 106 participants (77.9%) said that they would like anonymously to audit the professionals’ degree of compliance for at least 3 out of 4 safe practices.

Only the number of hospital visits was statistically different among participants who were willing to audit and those who were not in the bivariate analysis tests (Table 3). Consequently, the logistic regression only included the covariate ‘number of hospital visits’, with an associated Odds ratio (OR = 2.120 (95%CI: 1.467–3.065). Participants who had more visits were more willing to audit.

**Table 3:** Characteristics of P&Fs “willing” and “not willing” to become auditors as shown from the bivariate analysis

		Willing to audit (> = 3 safe practices)	Not willing to audit (< 3 safe practices)	p value
Age: mean (sd)				
		57.1 (14.1)	57.8 (12.8)	0.799
Gender: n (%)				
	Female	52 (72.2)	20 (27.8)	0.101
	Male	54 (84.4)	10 (15.6)	
Education: n (%)				
	Basic level	51 (78.5)	14 (21.5)	0.821
	Medium level	26 (81.2)	6 (18.8)	
	High level	27 (75.0)	9 (21.8)	
Type of participants: n (%)				
	Companions	36 (78.3)	10 (21.7)	0.949
	Patients	70 (77.8)	20 (22.2)	
Type of treatment: n (%)				
	Chemotherapy	80 (79.2)	21 (20.8)	0.637
	Transfusion	26 (74.3)	9 (25.7)	
Healthcare professional n (%)				
	Yes	10 (71.4)	4 (28.6)	0.509
	No	81 (78.6)	22 (21.4)	
Number of day hospital visits: mean (sd)				
		10.5 (9.6)	7.3 (5.9)	0.030
Number of hospital stays mean (sd)				
		1.03 (1.37)	0.9 (0.76)	0.217
Adverse events: n (%)				
	Yes	21 (70)	9 (30)	0.318
	No	85 (80.1)	21 (19.8)	
General perception of hospital safety: n (%)				
	Totally safe	51 (85)	9 (15)	0.075
	All other categories	55 (73.3)	20 (26.7)	

Among 104 participants who were willing to participate, 45 (43.3%) were “*good auditors*”. Of 63 participants categorized as *“good auditors”,* 45 (71.4%) were willing to audit.

## Discussion

It is known from research that there is a gap in professionals’ adherence to safe practices [6]. Qualitative studies have shown that patients are aware of the medication errors that are occurring and are prepared to participate actively in their prevention [22, 23]. Also, some works show that healthcare professionals, like patients, generally view patient involvement positively [24].These suggest that new methods are needed to assess professionals’ adherence to safe practices.

Some authors highlight that a video could be an important educational tool for increasing patients’ knowledge of the role they can play during hospitalization [25].

In this context, the study had the main objectives of finding out if patients and family members could learn the skills and were willing to audit safe practices anonymously.

In our study, sixty-three participants (46.3%) were classified as *“good auditors”* after the training. Younger age, high educational level, experience of an adverse event are the characteristics that best predict a P&Fs being a “good auditor”.

A recent Korean publication has shown similar results to ours when examining socio-demographic variables in a study over 600 individuals to analyse patient engagement with patient safety. Their results showed that older subjects and lower educational level participants felt less confidence to engage in patient safety activities [26].

Our study showed that P&Fs with higher education status, a proxy for health literacy [27], were better equipped to identify non-compliance with safety protocols, however this was not exclusive and, albeit a smaller proportion, some patients with basic education levels were also found to be good auditors (see Table 2).

We recruited all kinds of patients, so making simple and clear training materials (video and leaflets) was a requisite for us. In fact, 19 out of 48 volunteers (39.5%) who evaluated the material before the study had not completed higher education and after the debriefing, their feedback about the training materials was good. Training carried out based on videos and brochures may facilitate the acquisition of knowledge for people with certain characteristics. It would be necessary to explore whether another type of training allows them to achieve the same competencies regardless their age or education level.

In addition, in this study, relatives had better skills to play the role of the auditor in the bivariate analysis and they improved after the training more than patients did but the result did not reach statistically significant differences in the regression model, probably due to the relatives’ sample size.

Our research showed that more than 3 out of 4 participants were willing to play the role of the safety auditor. Several reasons can explain this finding, some of which are related to the process of reporting data to healthcare organizations. In the first place, we proposed that the assessment must be done anonymously so the participants would not have to confront healthcare professionals. Second, the participants received training and this induced more confidence in their skills. In fact, 89% participants answered that they knew how to assess safe practices after the training offered. Other reasons are related to the participants’ characteristics. Oncology patients may perceive a high risk of an error and thus be more willing to play an active role in patient safety. All these are enablers of patient involvement in patient safety [20, 28, 29]. In the study P&Fs’ participation was intended to simulate a continuous assessment during their process of care. Their willingness here may be different to a more conventional audit team, in which the P&F is a member along with healthcare professionals.

On the other hand, our research showed that the willingness to audit safe practices was different depending on the safe practice and whilst these differences did not reach statistical significance, it is interesting to note which practices were selected. Transfusion or chemotherapy identification were the safe practices that P&Fs were more willing to audit while hand hygiene was the least selected practice. There are many reasons that could influence P&Fs preference and willingness to engage in their healthcare. Some studies revealed that there is a general expectation that healthcare professionals, “know what they are supposed to be doing” and a common assumption that they always did what they were supposed to do, specially the most basic duties as washing their hands properly or administering the correct medications [14, 28–30]. Also, some studies suggest that checking to ensure that healthcare professionals were doing their job correctly could be embarrassing and damage relationship with them [20, 29].

Participants were offered training, including videos and reading material, before assessing their observation skills to increase their health literacy. Previous research has indicated that videos can be an important educational tool for increasing patients’ knowledge of the role they can play during hospitalization [25]. After the training, almost half of participants were considered to have the skills to be an auditor. It means that not every P&F willing to be auditor could or should be. Younger participants with high education level who have experienced an adverse event made the best auditors. Here we recommend that organizations develop methods to assess the skills of P&Fs before they are fully engaged in this audit process.

P&Fs’ assessment of gaps in safe practices gives the organization real-time data in order to engage them in the plan-do-check-act cycle. Furthermore, the fact that professionals may feel observed could encourage their adherence to safe practices.

Further research is needed a) to find ways to engage patients across the full range of age and educational levels, and b) to assess the full impact of this type of training in real situations, for example on patients and relatives willingness to question health carers about patient safety during their ongoing cancer treatment.

### Limitations

Although P&Fs as well as the environmental frame are real-world entities, the evaluation of the professionals’ safe practice adherence was undertaken by watching videos. To know the validity of involving P&Fs as auditors, it is necessary to compare the observations of P&Fs against a gold standard. The research project required an evaluation under controlled conditions because it was not possible to add additional observers to the P&Fs themselves in order to avoid the Hawthorne effect.

The evaluation of the role of the P&Fs as auditors of safe practices is an innovative approach. Therefore, from an ethical point of view, it seems more reasonable to assess safe practice under real conditions only if minimum guarantees of success are met. Further, the acceptance of the role of P&Fs as auditors implies, not only that the P&Fs themselves have accepted this function, but also that the healthcare professionals and the management team accept it. Using data to demonstrate that P&Fs are able to audit correctly facilitates the acceptance of this new role by health professionals and P&Fs.

## Conclusions

Using P&Fs as auditors of safe practices has many advantages. It goes beyond P&Fs giving an opinion or filling a perception questionnaire. Auditing goes directly to the quality assessment of healthcare organizations [29]. Using patients and family members in auditing allows for continuous monitoring and highlighting the importance of the inherent and essential agents in every healthcare process: the patient and the companion. Furthermore, it allows organizations to assess areas and departments that it would otherwise be impossible or that would require extraordinary efforts.

This new role has advantages not only for the organization but also for the P&Fs themselves. In order to play this new role P&Fs have to acquire the necessary skills. These new skills can enable them to adopt more active behaviours towards professionals such as “speaking up” and thus add new safety layers in providing a safer care [11].

Our research has shown that P&Fs are willing to play the safety auditor role and can be trained to perform this role. The research also highlights the characteristics of a good auditor and this will be of benefit to organizations that want to implement this strategy.

Patients’ participation in auditing safe care can be an innovative and viable approach to helping organisations improve the safety of the care they deliver.

## Data Availability

The datasets generated and/or analyzed during the current study are not publicly available but are available from the corresponding author on reasonable request via email: mi.rodrigo.rincon@cfnavarra.es

## Abbreviations

EPSF: European Patient Safety Foundation
P&Fs: Patients and their families
WHO: World Health Organization

## References

[1] Leavitt M (2001). Medscape's response to the Institute of Medicine Report: crossing the quality chasm: a new health system for the 21st century. MedGenMed..

[2] Kohn L, Corrigan JM, Donaldson M (2000). To err is human: building a safer health system.

[3] Zegers M, De Bruijne MC, Spreeuwenberg P, Wagner C, Van Der Wal G, Groenewegen PP (2011). Variation in the rates of adverse events between hospitals and hospital departments. Int J Qual Health Care.

[4] Edwards IR (2005). The WHO World Alliance for patient safety: a new challenge or an old one neglected?. Drug Saf.

[5] Lawton R, O'Hara JK, Sheard L (2017). Can patient involvement improve patient safety? A cluster randomised control trial of the patient reporting and action for a safe environment (PRASE) intervention. BMJ Qual Saf.

[6] Severinsson E, Holm AL (2015). Patients’ role in their own safety—a systematic review of patient involvement in safety. Open J Nurs.

[7] Norton PG, Baker GR (2007). Patient safety in cancer care: a time for action. J Natl Cancer Inst.

[8] Walsh KE, Dodd KS, Seetharaman K (2009). Medication errors among adults and children with cancer in the outpatient setting. J Clin Oncol.

[9] Hemoterapia UdHÁd (2019). Hemovigilancia. Año 2016. Subdirección General de Promoción de la Salud y Vigilancia en Salud Pública . Dirección General de Salud Pública, Calidad e Innovación.

[10] Davis RE, Vincent CA, Murphy MF (2011). Blood transfusion safety: the potential role of the patient. Transfus Med Rev.

[11] Longtin Y, Sax H, Leape LL, Sheridan SE, Donaldson L, Pittet D (2010). Patient participation: current knowledge and applicability to patient safety. Mayo Clin Proc.

[12] McGuckin M, Storr J, Longtin Y, Allegranzi B, Pittet D (2011). Patient empowerment and multimodal hand hygiene promotion: a win-win strategy. Am J Med Qual.

[13] Commission J (2019). Data of Sentinel Events Reviewed by The Joint Commission. Sentinel Events Reviewed by The Joint Commission 1995 Through 2018.

[14] Fisher KA, Smith KM, Gallagher TH, Huang JC, Borton JC, Mazor KM (2019). We want to know: patient comfort speaking up about breakdowns in care and patient experience. BMJ Qual Saf.

[15] Iedema R, Allen S, Britton K, Gallagher TH (2012). What do patients and relatives know about problems and failures in care?. BMJ Qual Saf.

[16] Fisher K, Smith K, Gallagher T, Burns L, Morales C, Mazor K (2017). We want to know: eliciting hospitalized Patients' perspectives on breakdowns in care. J Hosp Med.

[17] Ursprung R, Gray JE, Edwards WH (2005). Real time patient safety audits: improving safety every day. Qual Saf Health Care.

[18] Le-Abuyen S, Ng J, Kim S (2014). Patient-as-observer approach: an alternative method for hand hygiene auditing in an ambulatory care setting. Am J Infect Control.

[19] van Gelderen S, Hesselink G, Westert G, Robben P, Boeijen W, Zegers M. Optimal governance of patient safety: a qualitative study of barriers to and facilitators for effective internal audit. J Hosp Admin. 2017;6. 10.5430/jha.v6n3p15.

[20] Doherty C, Stavropoulou C (2012). Patients' willingness and ability to participate actively in the reduction of clinical errors: a systematic literature review. Soc Sci Med.

[21] Davis RE, Sevdalis N, Jacklin R, Vincent CA. An examination of opportunities for the active patient in improving patient safety. J Patient Saf. 2012;8(1):36–43. 10.1097/PTS.1090b1013e31823cba31894. 10.1097/PTS.0b013e31823cba94 22258225

[22] Schwappach DL, Wernli M (2010). Chemotherapy patients' perceptions of drug administration safety. J Clin Oncol.

[23] Schwappach DL, Wernli M (2010). Predictors of chemotherapy patients' intentions to engage in medical error prevention. Oncologist.

[24] Hrisos S, Thomson R (2013). Seeing it from both sides: do approaches to involving patients in improving their safety risk damaging the trust between patients and healthcare professionals? An interview study. PLoS One.

[25] Pinto A, Vincent C, Darzi A, Davis R (2013). A qualitative exploration of patients' attitudes towards the 'Participate inform notice Know' (PINK) patient safety video. Int J Qual Health Care.

[26] Lee HJ, Jang SG, Choi JE, et al. Assessment of Public Perception Regarding Patient Engagement for Patient Safety in Korea [published online ahead of print, 2019 Jan 10]. J Patient Saf. 2019; 10.1097/PTS.0000000000000565.doi:10.1097/PTS.0000000000000565. 10.1097/PTS.0000000000000565 PMC7781086 30633064

[27] Paasche-Orlow MK, Parker RM, Gazmararian JA, Nielsen-Bohlman LT, Rudd RR (2005). The prevalence of limited health literacy. J Gen Intern Med.

[28] Ringdal M, Chaboyer W, Ulin K, Bucknall T, Oxelmark L (2017). Patient preferences for participation in patient care and safety activities in hospitals. BMC Nurs.

[29] Davis RE, Jacklin R, Sevdalis N, Vincent CA (2007). Patient involvement in patient safety: what factors influence patient participation and engagement?. Health Expect.

[30] Carman KL, Dardess P, Maurer M (2013). Patient and family engagement: a framework for understanding the elements and developing interventions and policies. Health Aff (Millwood).

